# Metabolic surgery for type II diabetes: an update

**DOI:** 10.1007/s00592-021-01722-w

**Published:** 2021-05-18

**Authors:** Paolo Gentileschi, Emanuela Bianciardi, Domenico Benavoli, Michela Campanelli

**Affiliations:** 1 Department of Bariatric and Metabolic Surgery, San Carlo of Nancy Hospital, University of Rome Tor Vergata, Rome, Italy; 2 Department of Systems Medicine, Chair of Psychiatry, University of Rome Tor Vergata, Rome, Italy; 3 Department of Surgery, Policlinico Tor Vergata, Rome, Italy

**Keywords:** Metabolic surgery, Bariatric surgery, Laparoscopy, Diabetes, Obesity

## Abstract

Bariatric operations have been documented in clinical trials to promote remission or dramatic improvement of Type II Diabetes Mellitus and related comorbidities. Herein we review randomized trials and meta-analyses published during the last 20 years on the results of bariatric/metabolic surgery in obese patients with type 2 diabetes with the aim of highlighting the scientific evidence available. Several studies and RCTs in the last 20 years have showed outstanding results of bariatric/metabolic surgery on Type II diabetes and comorbidities in patients with either BMI > 35 kg/m^2^ or BMI < 35 kg/m^2^. They have established that bariatric procedures are superior to non-surgical interventions for inducing weight loss and amelioration of type 2 diabetes, even in patients with a BMI between 30 and 35 kg/m^2^. The physiopatologic changes that improve glucose homeostasis after bariatric surgery remain unclear but glycemic control is improved after sleeve gastrectomy, duodenal-jejunal bypass, Roux-en-Y gastric bypass, gastric banding, One Anastomosis Gastric Bypass, and biliopancreatic diversion. Nevertheless, it is suggested that the various gastrointestinal procedures may have different effects and mechanisms of action. Metabolic surgery will help integrate knowledge and multidisciplinary expertise to provide a combination of conservative and surgical treatments for Type II diabetes. These treatments must be considered as complementary options and not alternative strategies, with the same goal of controlling diabetes and achieving cure.

## Introduction

Morbid obesity is an epidemic disease with a prevalence of 7–10%, predicting to increase to 20% by 2025, determining a significant worsening of obesity comorbidities [1]. As a consequence patients with multiple comorbidities including type II diabetes will negatively influence health-care systems and life expectancy [2, 3].

Conservative treatments of obesity are not effective on a long-term follow-up. The success rate is even worse in severely obese patients with type 2 diabetes (T2DM) that will increase to 439 million by 2030 and to 650 million cases by 2040 [4]. Although the pharmacological approach to treat T2DM has expanded considerably, few patients are able to achieve and maintain optimal glycemic control in the long-term [5]. In the USA, only 52% of patients with T2DM maintain HbA1c < 7% and only 19% reach this goal with LDL < 5.6 mmol/l and blood pressure < 130/80 mmHg that are considered parameters to reduce cardiovascular mortality [6].

Bariatric surgery has been proven to determine greater weight loss in morbidly obese patients compared to intensive medical approaches [7–10]. In addition laparoscopic techniques have become safer in recent years [9, 11, 12]. Postoperative complications are rare and long-term results are encouraging [13–16].

Various clinical trials have demonstrated that bariatric procedures induce remission or amelioration of T2DM and other obesity-related comorbidities [9, 10, 13, 17, 18]. Experimental studies and clinical investigations provide scientific evidence that metabolic effects of bariatric surgery are partly independent of weight loss. [19, 20] The knowledge that these operations lead to type II Diabetes remission provided a rational to the new concept of metabolic surgery. The aim is therefore to treat metabolic syndrome and type II Diabetes also in patients with mild obesity [21, 22].

For this review article, a selective search (RCT trials, meta-analyses) of two databases (PubMed and the Cochrane Library) between 2000 and 2020 was conducted to investigate the results of metabolic surgery in patients with T2DM and obesity with the aim of highlighting the robust scientific evidence arising from the latest literature works.

## Clinical trials

In the last twenty years, several studies have clearly documented results of bariatric/metabolic surgery on T2DM and associated comorbidities in patients with either BMI > 35 kg/m^2^ or BMI < 35 kg/m^2^ [23–25]. In the current literature review, we selected the improvement of type II diabetes as a criteria for the goal of metabolic surgery.

An overall 77% remission rate in a mean of 14.6% months of follow-up (range, 6–36) has been reported in 2004 in a meta-analysis of 22,094 diabetic patients by Buchwald et al. [23]. Unfortunately the meta-analysis was based mainly on retrospective studies with short follow-up. A large multicenter prospective study (Swedish Obese Subjects study) [24] compared different bariatric operations with conservative management in groups of well matched obese patients. Seventy-two percent of surgical patient experienced remission of type II diabetes versus 21% in the medically treated group at two years follow-up. The relative risk (RR) of type II diabetes was three times lower, and the rates of recovery three times greater for surgical patients compared with control group patients after 10 years. In this study, remission was marked by blood glucose < 100 ml/dl, and no diabetes medication needed.

More recently Gill et al. reviewed 27 studies on sleeve gastrectomy with 673 patients and reported a T2DM resolution rate of 66.2% in obese individuals with improved glycemic control in 26.9% [25].

Efficacy of bariatric and metabolic surgery has been showed also in several randomized controlled trials (RCT) comparing medical versus surgical management of T2DM. The Diabetes Surgery Study (DSS) randomized trial involved 120 patients to receive intensive lifestyle and medical therapy with or without RYGB [26]. The primary endpoint included achievement of HbA1c < 7.0%, LDL-cholesterol < 100 mg/dL, and systolic blood pressure < 130 mmHg. After 12 months, 28 participants in the RYGB group (49%, 95% CI 36–63%) and 11 in the medical management group (19%, 95% CI 10–32%) achieved the primary end points.

The STAMPEDE trial compared medical therapy alone versus medical therapy and Roux-en-Y gastric bypass (RYGB) or Sleeve Gastrectomy (SG) in 150 obese patients (BMI 27–43 kg/m^2^) with uncontrolled T2DM. The percentage of patients who reached the primary endpoint (HbA1c of 6.0% at 1 year) was 12% in the medical group, 42% in the RYGB group, and 37% in the SG group [27]. Also secondary end points, including BMI, body weight, waist circumference, and HOMA-IR were reported to improve after surgery.

Mingrone et al. reported the two years results of RCT in 2012 based on 60 diabetics patients (BMI > 35 kg/m^2^) of at least 5 years duration with an HbA1c level of > 7% comparing traditional medical therapy to RYGB and BPD. No patient in medical therapy group experienced diabetes remission versus 35% in the RYGB group and 95% in the BPD group [28]. The Lancet in 2015 published long-term results from this study [29]. Overall, 19 (50%) of the 38 patients maintained diabetes remission at 5 years compared with none of the 15 patients in the medically treated group (*p* = 0.0007). Hyperglycemia resolution occurred in 53% of the patients who achieved 2-year remission in the RYGB group and 37% of the patients in the BPD group. Weight changes did not predict diabetes that remission is relapse after surgery even if surgical patients lost more weight than the control group.

Until 2020, published literature data about long-term relapse of T2DM after metabolic surgery were limited. No RCTs of metabolic surgery for diabetes were available beyond 5 years of follow-up. In January 2021, Mingrone et al. reported the results of a 10-year follow-up study in the Lancet [30]. It’s the same study mentioned above but with a longer-term evaluation of 60 patients with a follow-up rate of 95.0%. Ten-year remission rate were 5.5% for medical therapy (95% CI 1.0–25.7), 50.0% for BPD (29.9–70.1), and 25.0% for RYGB (11.2–46.9; *p* = 0.0082). All individuals with relapse maintained adequate glycemic control at 10 years. Participants in the surgical groups had fewer diabetes-related complications than those in the medical therapy group. Authors conclude that metabolic surgery is more effective than conventional medical therapy in the long-term control of T2DM. The importance and results of this study should advice clinicians and policy makers to ensure that metabolic surgery is considered in the clinical treatment of patients with obesity and T2DM.

Also for individuals with T2DM and BMI < 35 kg/m^2^ clinical studies and RCTs report with a robust evidence that this group of patients may benefits in the disease control from metabolic surgery. A RCT by Dixon et al. in 2008 enrolled individuals with BMI 30–35 kg/m^2^ comparing gastric banding with medical treatment [31]. The authors showed at 2-year follow-up remission of T2DM in 73% in the surgical group and 13% in the medical therapy group.

In 2012, a prospective study of 66 diabetic patients with BMI 30–35 kg/m^2^ who submitted to RYGB showed remission of diabetes in 90% of them after a follow-up of 6 years [32]. Positive results were also seen in the DSS trial [26] including less obese patients with diagnosis of T2DM and follow-up of 12 months. Surgery was associated with greater improvement in HbA1c, LDL-cholesterol, and blood pressure that with medical treatment.

In a recent study, 1016 consecutive patients underwent metabolic surgery with a minimum 1 year follow-up, and the authors reported a significantly higher 1-year total weight loss (30.5%) and type 2 diabetes mellitus (T2DM) remission (78.4%) in the OAGB group [33].

Recently, Soong and Colleagues showed long-term outcomes of 134 OAGB patients for the treatment of T2DM with 5 years follow-up. The complete T2DM remission rate of OAGB was 76.1% at 1 year and 64.2% at 5 years after surgery, while the T2DM recurrence rate was 15.7%. Finally, forty-one (57.8%) out of 71 patients who completed a 10-year follow-up remained in complete T2DM remission [34].

A recent meta-analysis of published studies [35] reporting on diabetes control after bariatric/metabolic surgery considered 94,579 surgical patients. The authors collected all reports appeared in the literature till then. The meta-analysis showed that improvement rates were equivalent in the 60 investigations in which mean baseline BMI of the study cohorts was > 35 kg/m^2^ and the 34 studies including patients with mean baseline BMI < 35 kg/m^2^ (71% vs. 72%, respectively).

Another recent meta-analysis and systematic review of RCTs focused on two bariatric procedures, Laparoscopic RYGB and Sleeve Gastrectomy for T2DM in non-severely obese patients with BMI < 35 kg/m^2^ [36]. Four RCTs concerning total 296 patients were included. T2DM remission and %EWL were similar between the two procedures but both operations improved substantially BMI, waist circumference, LDL, HbA1c, fasting plasma glucose, total cholesterol, and triglyceride.

Considering these outstanding results of metabolic surgery in this group of patients, the indication for the surgical approach to T2DM with mild obesity should be fully considered by clinical Institutions all over the world. Furthermore, surgery in patients with BMI < 35 kg/m^2^ should be even safer than bariatric surgery given the less complex clinical features of this group of patients. In Table 1, the results of our literature review concerning mechanisms of action of most popular procedures are summarized. The physiopatologic changes that improve diabetes control after metabolic surgery remain unclear but glucose homeostasis is improved after sleeve gastrectomy (SG), duodenal-jejunal bypass (DJB),Roux-en-Y gastric bypass (RYGB), gastric banding, One Anastomosis Gastric Bypass (OAGB), and biliopancreatic diversion (BPD). Nevertheless, it is also plausible that different gastrointestinal surgeries may have distinct effects and mechanisms of action (Table 2).

**Table 1:** Mechanisms of action of most popular procedures

Type of procedure	Mechanism of action	References
	Changes in gut hormones (↓ghrelin, ↑GLP-1, ↑insulin, ↑post-prandial PYY and oxyntomodulin levels)	Gill RS, Birch DW, Shi X. Surg Obes Relat Dis. 2010 [25] Sha Y, Huang X, Ke P. Obes Surg. 2020 [36]
	Changes in gut hormones (↓ghrelin, ↑GLP-1, ↑insulin, ↑post-prandial PYY and oxyntomodulin levels) ↑glucose metabolism, changes in bile acid signaling Changes in nutrient sensing improving insulin sensitivity ↓glucose transport in the intestine (SGLT1) ↓circulating aminoacids Changes in gut microbiota	Rubino F, Gagner M, Gentileschi P. Ann Surg. 2004 [21] Ikramuddin S, Korner J, Lee WJ. JAMA. 2013 [26] Batterham RL, Cummings DE. Diabetes Care 2016 [39] Cummings DE, Rubino F. Diabetologia. 2018 Feb [4]
	Changes in gut hormones (↓ghrelin, ↑GLP-1, ↑insulin, ↑post-prandial PYY and oxyntomodulin levels) ↑glucose metabolism, changes in bile acid signaling Changes in nutrient sensing improving insulin sensitivity ↓glucose transport in the intestine (SGLT1) ↓circulating aminoacids Changes in gut microbiota	Rubino F, Gagner M, Gentileschi P. Ann Surg. 2004 [21] Batterham RL, Cummings DE. Diabetes Care 2016 [39] Cummings DE, Rubino F. Diabetologia. 2018 Feb [4] Mingrone G, Panunzi S, De Gaetano A. Lancet. 2021[30]

The molecular mechanisms that improve glycemic control after metabolic surgery remain unclear. In spite of anatomical and functional differences between procedures, glucose homeostasis is improved after all these type of procedures. Nevertheless, it is also plausible that different gastrointestinal surgeries may have distinct effects and mechanisms of action

**Table 2:** High Quality Clinical trials published in literature between 2004 and 2021

Authors	N° of cases	Type of surgery	Overall remission rate	Follow-up
Buchwald [23]	22,094	Gastric Banding 47.5% (40.7–54.2%) Gastric Bypass 61.6% (56.7–66.5%) Gastroplasty 68.2% (61.5–74.8%) Biliopancreatic Diversion Or Duodenal Switch. 70.1% (66.3–73.9%)	77%	14.6 months (range 6–36)
Sjöström [24]	343	Adjustable or non-adjustable banding(*n* = 61), Vertical banded gastroplasty (*n* = 227), Gastric bypass (*n* = 55)	30.4%	10 years
Gill [25]	673	SG	66.2%	13.1 months (range 3–36)
Ikramuddin [26]	60	RYGB	95%	12 months
Schauer [27]	97	RYGB	RYGB 42% SG 37%	3 years
Mingrone [28]	40	RYGB 20 BPD 20	RYGB 75%; BPD95%	2 years
Mingrone [29]	40	RYGB 20 BPD 20	RYGB 37% BPD 63%	5 years
Mingrone [30]	40	RYGB 20 BPD 20	BPD 50.0%; RYGB 25.0%	10 years
Dixon [31]	30	Laparoscopic gastric banding	73%	2 years
Cohen [32]	66	RYGB	88%	5 years [range 1–6]
Panunzi [33]	4944	Laparoscopic gastric banding, RYGB, sleeve Gastrectomy, duodenal-jejunal bypass (DJB), and Biliopancreatic diversion (BPD)	PBD 89%, RYGB77%, gastric banding 62%, SG 60%	> 6 months
Shen [34]	1016	RYGB 197, OAGB 171, SG 437, SG-DJB 130, SA-DJBSG 81	78.4%	1 year
Soong [35]	134	OAGB	76.1% (1 year) 64.2% (5 years) (57.8%) out of 71 10-year	10 years
Sha [36]	296	RYGB 151 SG 145	RYGB 54.0%, SG 56.7%	24 to 60 months

*SG* Sleeve Gastrectomy; *RYGB* Roux-en-Y gastric Bypass; *OAGB* One Anastomosis Gastric Bypass; *BPD* Biliopancreatic Diversion; *SG-DJB* SG with Duodenal-Jejunal Bypass; *SA-DJBSG* Single Anastomosis Duodenal-Jejunal Bypass with SG

## A new era

High quality data from RCTs have clearly established that bariatric procedures are more effective than medical or lifestyle interventions for inducing weight loss and remission of type 2 diabetes, even in mild obese patients with a BMI between 30 and 35 kg/m^2^. The second Diabetes Surgery Summit (DSS-II) introduced new guidelines that recommend consideration of metabolic surgery among anti-diabetic interventions for selected patients with obesity and T2DM [37]. As a result, in 2017 DSS-II guidelines were incorporated into the ADA Standards of Diabetes Care [38]. Metabolic surgery, including bariatric procedures previously performed for severe obesity, is now considered as a standard diabetes treatment option for selected individuals with poorly controlled T2DM and BMI > 30 kg/m^2^.

The exact physiopatologic mechanism responsible of improved glycemic control after bariatric surgery is unclear although most popular procedures have anatomical and functional difference.

Glucose homeostasis is improved after all types of operation, probably as an expression of weight loss and partial overlap in the mechanism of action. However, given the specific physiological role of the stomach and various intestinal segments in regulating glucose homeostasis, it is also plausible that different gastrointestinal surgeries may have distinct effects and mechanism of action. Although much research has been published and the exact pathway to diabetes improvement remains unidentified, the scientific evidence from RCTs on metabolic surgery for T2DM and obesity is strong, and new guidelines have been proposed (DSS-II) [39].

Metabolic surgery reflex integration of scientific knowledge and multidisciplinary expertise to offer patients a combination of medical and surgical treatment. These are not alternative but complementary with a final goal based on controlling diabetes and achieving cure.
